# Cigarette smoke extract-induced p120-mediated NF-κB activation in human epithelial cells is dependent on the RhoA/ROCK pathway

**DOI:** 10.1038/srep23131

**Published:** 2016-09-02

**Authors:** Chao Zhang, Shenghui Qin, Lingzhi Qin, Liwei Liu, Wenjia Sun, Xiyu Li, Naping Li, Renliang Wu, Xi Wang

**Affiliations:** 1 Institute of Pathology, Tongji Hospital, Tongji Medical College, Huazhong University of Science and Technology, Wuhan 430030, China

## Abstract

Cigarette smoke exposure is a major cause of chronic obstructive pulmonary disease (COPD), but the underlying molecular inflammatory mechanisms remain poorly understood. Previous studies have found that smoke disrupts cell-cell adhesion by inducing epithelial barrier damage to the adherens junction proteins, primarily E-cadherin (E-cad) and p120-catenin (p120). Recently, the anti-inflammatory role of p120 has drawn increasing attention. In this study, we demonstrate that p120 has a role in the cigarette smoke extract-induced inflammatory response, presumably by regulating NF-κB signaling activation. Mechanistically, we show that p120-mediated NF-κB signaling activation in airway epithelial inflammation is partially RhoA dependent and is independent of E-cad. These results provide novel evidence for the role of p120 in the anti-inflammatory response.

Cigarette smoking is a major cause of chronic obstructive pulmonary disease (COPD), which is one of the most important causes of morbidity and mortality in the world. COPD is characterized by the destruction of lung tissue and the loss of alveolar cells after inflammation^1^. Epithelial barrier function is the first line of defense against inhaled noxious insults. A persistent increase in airway epithelial permeability occurs in smokers and individuals exposed to secondhand smoke^2^,^3^ where the epithelial barrier is disrupted and the subepithelial tissue gets directly exposed to reactive chemicals and oxidants/free radicals^4^,^5^. This insult triggers an inflammatory response that, if sustained, can lead to structural epithelial abnormalities such as remodeling and mucous metaplasia^6^; both of which are hallmarks of COPD and asthma. Despite the pervasiveness of COPD, the precise pathophysiological mechanism is not well described.

Adherens junctions (AJs) modulate cell-cell adhesion between epithelial cells through complexes that are composed of E-cadherin (E-cad), p120-catenin (p120), β-catenin (β-ctn), and α-catenin (α-ctn)^7^. Smoke disrupts cell-cell adhesions and damages the epithelial barrier via the AJ proteins, including E-cad and p120 8,9. p120 is a member of a subfamily of armadillo (ARM) repeat-containing proteins, which are known to regulate E-cadherin-mediated cell-cell adhesion^10^. Emerging evidence indicates that p120 acts as an endogenous anti-inflammatory mediator in many tissues and organs^11^,^12^,^13^,^14^. Conditional p120 knockout mice exhibited increased inflammatory cell infiltration in a tissue-specific ablation of p120 by invoking the NF-κB signaling pathway and the production of proinflammatory cytokines even in the absence of infection^11^,^12^. The expression of p120 in endothelial cells modulates endotoxin-induced lung inflammation by interfering with NF-κB signaling^15^. Our previous research showed that p120 acts as an anti-inflammatory mediator in LPS-stimulated and mechanical scratch-stimulated bronchial epithelial cells (BECs) by modulating NF-κB signaling^16^,^17^,^18^. However, the mechanism by which p120 modulates NF-κB signaling in airway inflammation has not yet been fully elucidated.

RhoA is the most well studied member of the Rho GTPases and is the master regulator of actin dynamics. Its highly localized activity is required to induce proper cell polarity, migration, AJ formation and maturation^19^. p120 directly regulates RhoA activity by functioning as a Rho GDI to sequester Rho-GDP^20^. Recently, research has shown that RhoA also plays an important role in the inflammatory response. The GEF-H1-RhoA signaling pathway mediates LPS-induced NF-κB transactivation and IL-8 synthesis in endothelial cells^21^. The RhoA/ROCK pathway might regulate NF-κB activity to up-regulate inflammatory genes and mediate the development of diabetic nephropathy^22^. The blockade of ROCK inhibits the activation of NF-κB and the production of proinflammatory cytokines, suggesting a critical role for ROCK in the synovial inflammation of rheumatoid arthritis^23^. Rho kinase is upstream of NF-κB in driving LPA-mediated adhesion molecule expression^24^. As noted above, p120 and RhoA/ROCK are shown to be involved in inflammation and inflammation-related diseases; therefore, we hypothesize that p120 might also play an important role in cigarette smoke extract (CSE)-induced airway injury through the NF-κB signaling pathway via the RhoA/ROCK axis.

## Results

### p120 expression was down-regulated after exposure to CSE at different concentrations and time points

**Figure 1:**
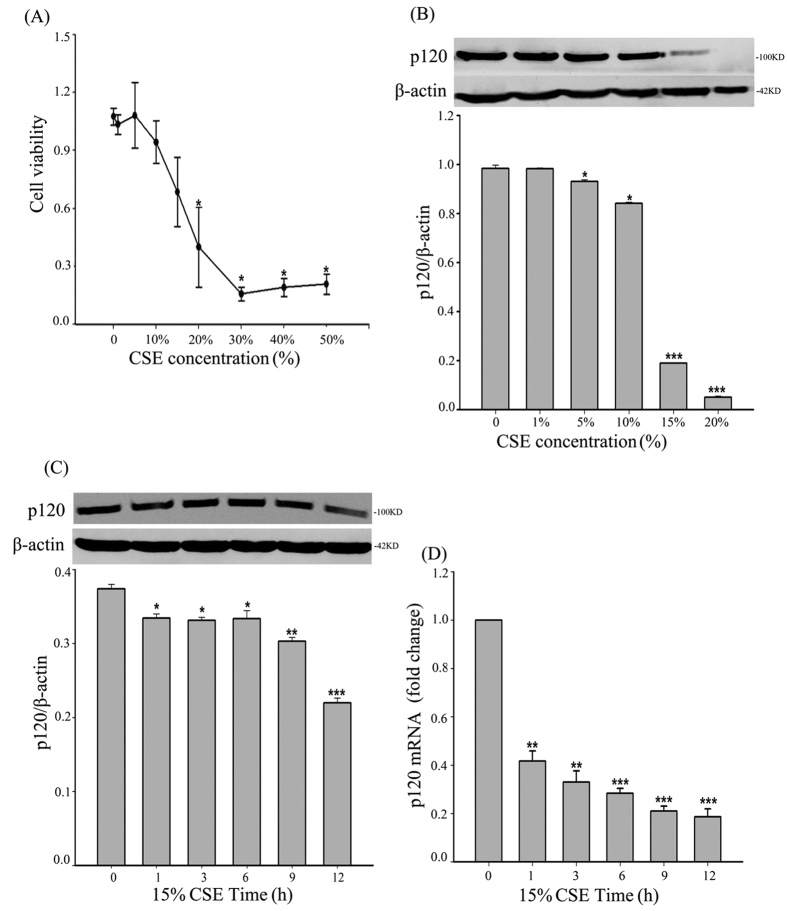
p120 was down-regulated after CSE stimulation. (**A**) The cytotoxic effect of CSE on the 16HBE cells was assessed by an MTT assay. The data represent the means from three independent experiments and were analyzed by a one-way ANOVA test. ^*^P < 0.05 vs. the control group. (**B**) The cells, after treatment with different concentrations of CSE, were analyzed by western blot to detect p120. β-actin was the loading control. The data were expressed as the means ± SD (n = 3), ^*^P < 0.05, and ^***^P < 0.001 vs. the control group. (**C**) After CSE exposure for different time points, the cells were analyzed by western blot to detect p120. β-actin was the loading control. The data were expressed as the means ± SD (n = 3), ^*^P < 0.05, ^**^P < 0.01, and ^***^P < 0.001 vs. the control group (0 h). (**D**) After CSE stimulation, total mRNA of p120 was extracted at the indicated times. The columns represented the relative amplification fold change of p120 in contrast to GAPDH. The data were expressed as the means ± SD (n = 3), ^**^P < 0.01, and ^***^P < 0.001 vs. the control group (0 h).

To investigate the effects of CSE on cell viability, a cytotoxicity assay was performed after 16HBE cells were exposed to different concentrations of CSE for 24 h. The MTT analysis revealed a dose-dependent reduction in cell viability. The cells remained approximately 80% viable at 15% CSE and had only approximately 40% viability at 20% CSE (Fig. 1A). Hence, six concentrations of CSE (0%, 1%, 5%, 10%, 15%, 20%) were chosen to study the effects of CSE on p120 expression in epithelial cells. After exposure to different concentrations of CSE for 24 h, a western blot showed that the expression of p120 gradually decreased as the concentration of CSE increased, and this effect was most obvious at 15% (Fig. 1B). Therefore, 15% CSE, which was the maximum concentration that did not cause a significant change in cell viability, was chosen for the following experiments.

After exposure to 15% CSE, a western blot revealed that p120 expression was reduced in a time-dependent manner (Fig. 1C). An RT-PCR analysis also indicated that p120 mRNA decreased in a time-dependent manner (Fig. 1D).

### NF-κB signaling pathway was activated after CSE stimulation in 16HBE cells

**Figure 2:**
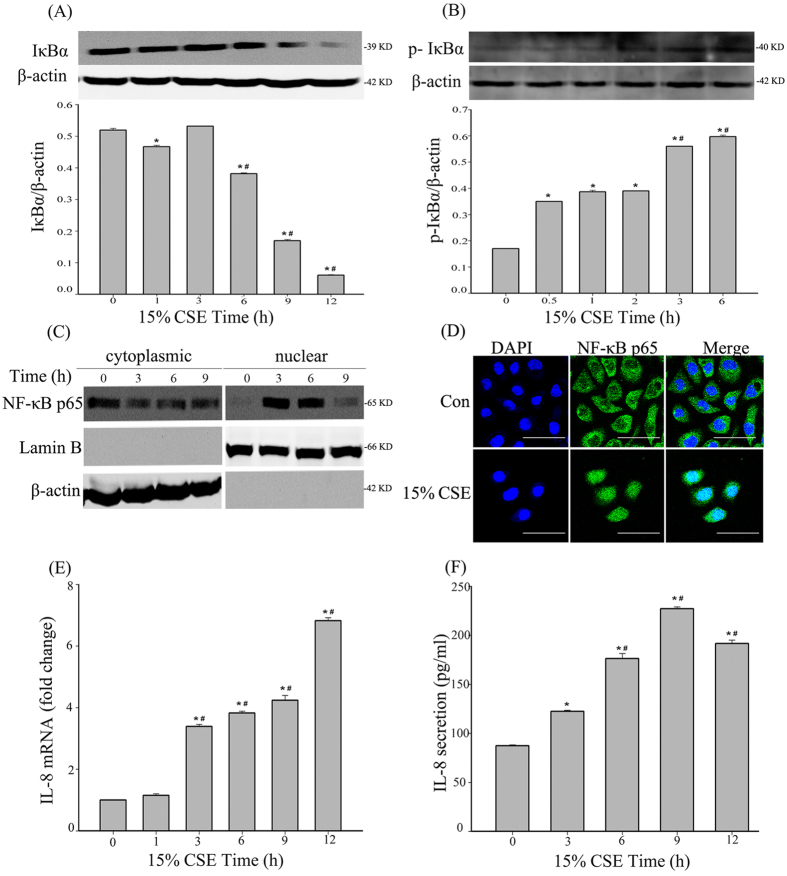
NF-κB signaling pathway is activated after CSE stimulation in 16HBE cells. (**A**) After 15% CSE exposure for different time point, the cells were analyzed by western blot to detect IκBα. β-actin was the loading control. The histograms show the ratio between IκBα and β-actin of 3 experiments. ^*^P < 0.05 vs. 0 h group, ^#^*P *< 0.05 vs. 1 h group. (**B**) After 15% CSE exposure for different time point, the cells were analyzed by western blot to detect phosphorylated IκBα (p-IκBα). β-actin was the loading control. The histograms show the ratio between p-IκBα and β-actin of 3 experiments. ^*^P < 0.05 vs. 0 h group, ^#^*P *< 0.05 vs. 0.5 h group. (**C**) The nuclear translocation of p65 after CSE exposure in the 16HBE cells. The cells were exposed to15% CSE for the indicated times before fractionation into cytoplasmic and nuclear extracts. β-actin and Lamin B were the loading controls for cytoplasmic and nuclear fractions, respectively. The quantified results can be found as Supplementary Fig. S1. (**D**) Fluorescence staining of NF-κB-p65. After 6 h of CSE exposure, cells grown on coverslips were subjected to immunofluorescence staining. Scale bar, 50 μm. (**E**) After exposure to 15% CSE, IL-8 mRNA was measured by RT-PCR at the indicated times. The data were expressed as the means ± SD (n = 3), ^*^P < 0.05 vs. 0 h group, ^#^*P *< 0.05 vs. 1 h group. (**F**) The IL-8 concentration was measured by ELISA. The data were expressed as the means ± SD (n = 3), ^*^P < 0.05 vs. 0 h group, ^#^*P *< 0.05 vs. 1 h group.

The activation of NF-κB was assessed in 16HBE cells after exposure to 15% CSE at different time points. First, we found that the NF-κB static binding protein IκBα decreased (Fig. 2A), which was accompanied by an increase in p-IκBα (Fig. 2B) after the cells were exposed to 15% CSE for 12 h. Next, immunofluorescence and nuclear/cytoplasm protein extraction experiments were used to directly confirm the NF-κB-p65 activation. We found obvious p65 translocation into the nucleus and a decrease in cytoplasmic p65 (Fig. 2C,D, Supplementary Fig. S1). We also found a dramatic increase in proinflammatory cytokines at both the mRNA (Fig. 2E) and the protein levels (Fig. 2F) after CSE exposure.

### CSE-induced NF-κB activation was inhibited by p120 3A over-expression in 16HBE cells

**Figure 3:**
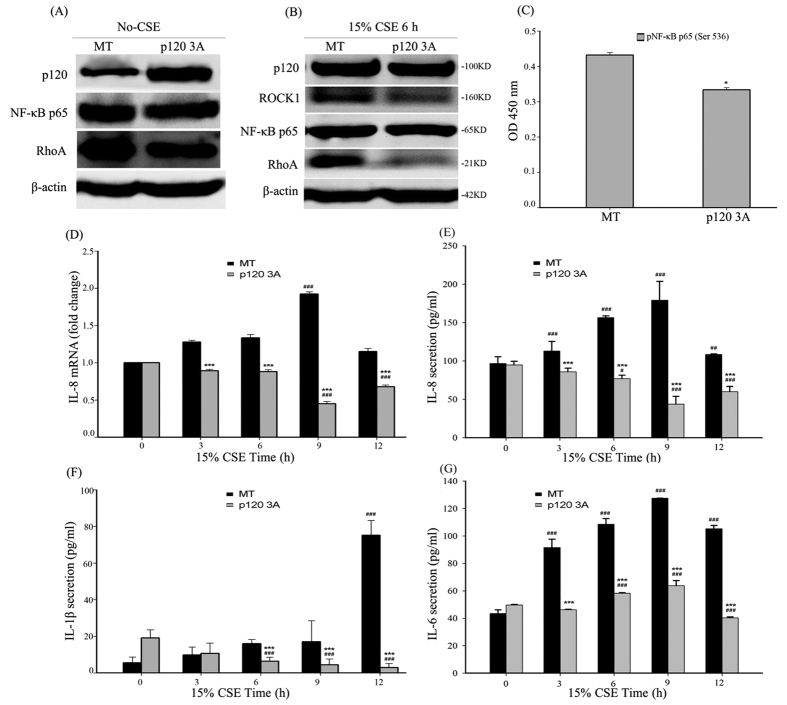
CSE-induced NF-κB activation is inhibited by the over-expression of p120 3A in 16HBE cells. The cells were transiently transfected with p120 3A or an empty vector control (mock transfection, MT) for 48 h. (**A**) Whole-cell protein was extracted and analyzed by western blot to detect p120, NF-κB-p65 and RhoA. β-actin was the loading control. The quantified results can be found as Supplementary Fig. S2A. (**B**) After 6 h of CSE exposure, the cells were analyzed by western blot to detect p120, ROCK1, NF-κB-p65 and RhoA 48 h after transfection. β-actin was the loading control. The quantified results can be found as Supplementary Fig. S2B. (**C**) After 6 h of CSE exposure, the cells were measured to assess the phosphorylation of NF-κB-p65 (Ser 536) by ELISA. The data are expressed as the means ± SD (n = 3), ^*^P < 0.05 vs. the MT group. (**D**) IL-8 mRNA was measured by RT-PCR. The data are expressed as the means ± SD (n = 3), ^***^P < 0.001 vs. the MT + CSE groups, ^###^*P *< 0.001 vs. the CSE 0 h group. (**E**–**G**) IL-8, IL-1β and IL-6 protein was examined by ELISA. The data are expressed as the means ± SD (n = 3), ^***^P < 0.001 vs. the MT + CSE groups, ^#^*P *< 0.05, ^##^*P *< 0.01, ^###^*P *< 0.001 vs. the CSE 0 h group.

To investigate the effects of p120 on the activation of NF-κB signaling by smoke, we transfected the plasmid RcCMVp120 3A into 16 HBE cells to observe the effects on NF-κB activity. The transfection efficiency was evaluated by western blot (Fig. 3A, Supplementary Fig. S2A). The over-expression of p120 3A had no obvious effect on the total NF-κB-p65 expression regardless of CSE exposure (Fig. 3A,B, Supplementary Fig. S2). However, using ELISA, we found that the phosphorylation of NF-κB-p65 (Ser536) was attenuated after 6 h of CSE exposure in the p120 3A over-expression group (Fig. 3C). Moreover, RT-PCR showed that IL-8 mRNA was down-regulated in the p120 3A over-expression group after CSE exposure for different time points (Fig. 3D). Additionally, the secretion of IL-8, IL-1β and IL-6 was decreased in the p120 3A over-expression group after CSE exposure for different time points as measured by ELISA (Fig. 3E–G).

### Knockdown of p120 enhanced CSE-induced NF-κB activation in 16HBE cells

**Figure 4:**
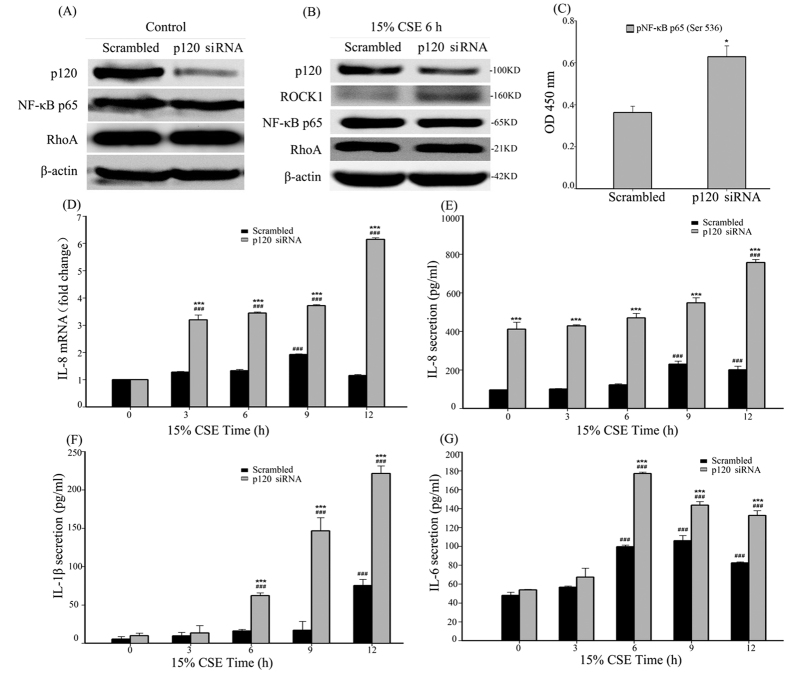
Knockdown of p120 enhances CSE-induced NF-κB activation in 16HBE cells. The cells were transfected with a p120 siRNA or a scrambled siRNA sequence for 48 h, (**A**) p120, NF-κB-p65 and RhoA expression were analyzed by western blot. β-actin was the loading control. The quantified results can be found as Supplementary Fig. S3A. (**B**) After 6 h of CSE exposure, the cells were analyzed by western blot to detect p120, ROCK1, NF-κB-p65 and RhoA. β-actin was the loading control. The quantified results can be found as Supplementary Fig. S3B. (**C**) After 6 h of CSE exposure, the cells were measured to assess the phosphorylation of NF-κB-p65 (Ser 536) by ELISA. The data are expressed as the means ± SD (n = 3), ^*^P < 0.05 vs. the Scrambled group. (**D**) IL-8 mRNA was measured by RT-PCR. The data are expressed as the means ± SD (n = 3), ^***^P < 0.001 vs. the Scrambled + CSE groups, ^###^*P *< 0.001 vs. the CSE 0 h group. (**E**–**G**) IL-8, IL-1β and IL-6 protein was examined by ELISA. The data are expressed as the means ± SD (n = 3), ^***^P < 0.001 vs. the Scrambled + CSE groups, ^###^*P *< 0.001 vs. the CSE 0 h group.

We further examined NF-κB activation after p120 knockdown in 16HBE cells. A western blot showed that p120 expression was significantly knocked down by siRNA (Fig. 4A, Supplementary Fig. S3A). The total NF-κB-p65 was not changed in both the scrambled and the p120-knockdown group after CSE exposure (Fig. 4A,B, Supplementary Fig. S3), but phospho-NF-κB-p65 (Ser536) was increased significantly in the p120-knockdown group after 6 h of exposure (Fig. 4C) compared with the scrambled group. At the same time, RT-PCR showed an increased up-regulation of IL-8 in the p120-knockdown group after CSE exposure for different time points (Fig. 4D). Meanwhile, the up-regulation of IL-8, IL-1β and IL-6 was enhanced in the p120-knockdown group after CSE exposure for different time points as measured by ELISA (Fig. 4E–G).

### CSE-induced NF-κB activation was regulated through the RhoA/ROCK axis in 16HBE cells

**Figure 5:**
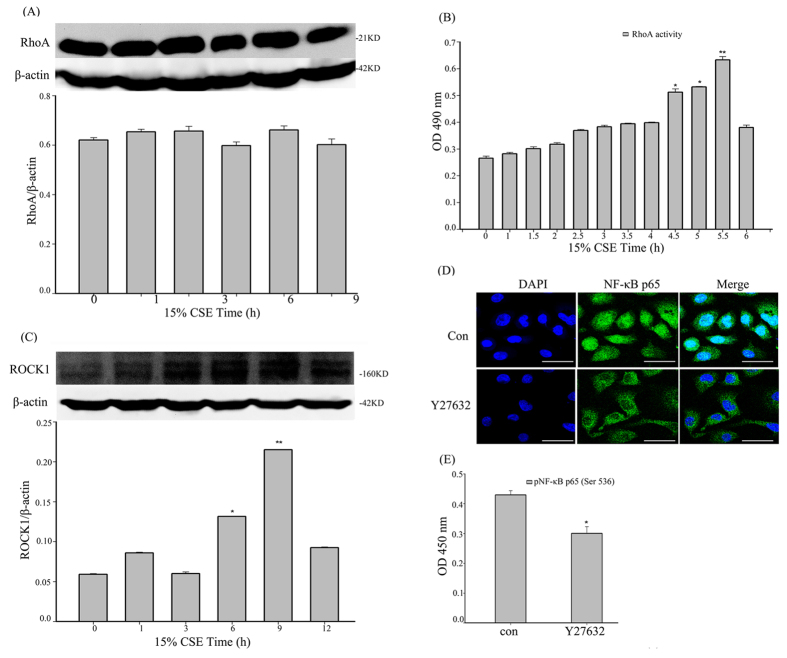
CSE-induced NF-κB activation is regulated through the RhoA/ROCK axis in 16HBE cells. (**A**) After CSE exposure, the cells were analyzed by western blot to detect RhoA. β-actin was the loading control. The data were expressed as the means ± SD (n = 3). (**B**) The G-LISA analysis was used to detect the active RhoA after CSE exposure at different time points. The data were expressed as the means ± SD (n = 3). ^*^P < 0.05, ^**^P < 0.01 vs. the control group (0 h). (**C**) After CSE exposure, the cells were analyzed by western blot to detect ROCK1. β-actin was the loading control. The data were expressed as the means ± SD (n = 3). ^*^P < 0.05, ^**^P < 0.01 vs. the control group (0 h). (**D**) The ROCK inhibitor Y27632 inhibited CSE-induced NF-κB activation. The cells were pretreated with 20 μM Y27632 or DMSO (solvent control) for 12 h before CSE stimulation (6 h). The nuclear translocation of p65 was detected by immunofluorescent staining. Scale bar, 50 μm. (**E**) After CSE exposure, the cell lysates were measured to detect the phosphorylation of NF-κB-p65 (Ser 536) by ELISA. The data are expressed as the means ± SD (n = 3), ^*^P < 0.05 vs. the control group.

The RhoA/ROCK pathway might regulate NF-κB activity to up-regulate inflammatory genes and mediate the development of diabetic nephropathy^22^. Therefore, we explored whether the RhoA/ROCK axis can also regulate NF-κB activation during airway inflammation in 16HBE cells. By western blot, we found the levels of total RhoA had not changed (Fig. 5A), but G-LISA showed that RhoA activity dramatically increased after 4.5 h of CSE exposure (Fig. 5B). Moreover, ROCK1, a protein downstream of RhoA, increased after 15% CSE exposure (Fig. 5C).

The ROCK inhibitor Y27632 was used to further investigate the association between RhoA and NF-κB. Before CSE exposure, the cells were pretreated with 20 μM Y27632 for 12 h to inhibit the RhoA pathway^22^. Immunofluorescence staining revealed that NF-κB-p65 nuclear translocation decreased significantly after 6 h of CSE stimulation (Fig. 5D), and the ELISA results also showed that the phosphorylation of NF-κB-p65 (Ser536) decreased in the Y27632 group (Fig. 5E).

### p120-regulated NF-κB activation occurs partially through the RhoA/ROCK axis in human bronchial epithelial cells after CSE exposure

**Figure 6:**
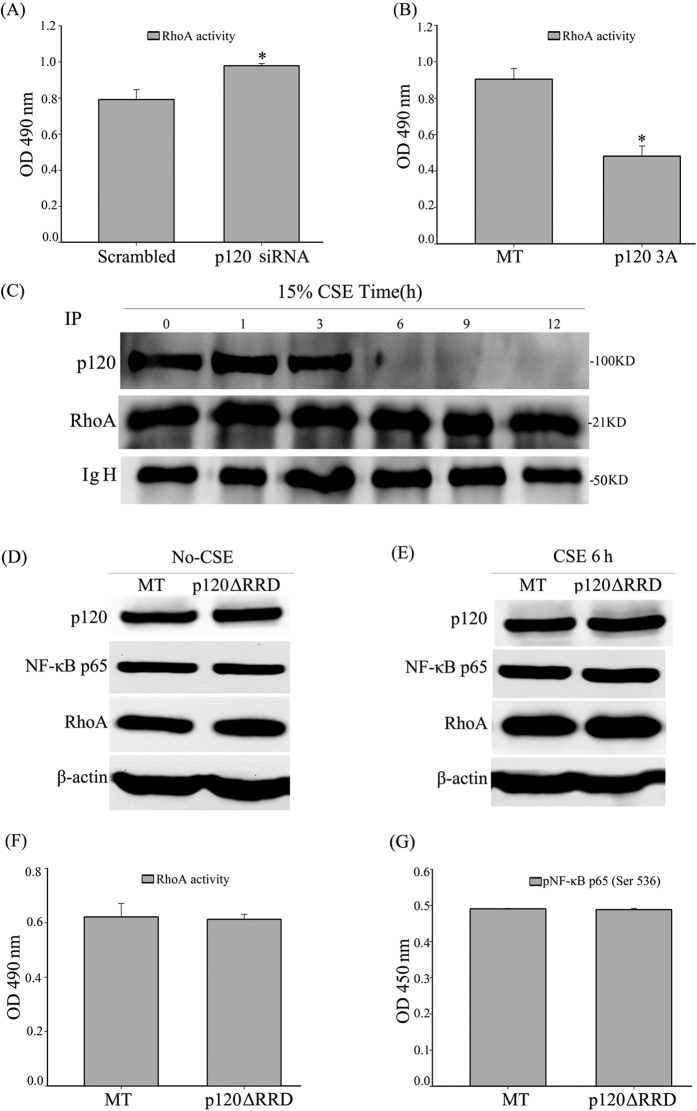
p120-meditaed NF-κB activation occurs partially through the RhoA/ROCK axis in human bronchial epithelial cells after CSE exposure. (**A**) RhoA activity in the scrambled and p120 siRNA groups after 15% CSE exposure was assessed by G-LISA. The data are expressed as the means ± SD (n = 3), ^*^P < 0.05 vs. Scrambled + CSE group. (**B**) RhoA activity in the MT and the p120 3A over-expression groups after 15% CSE exposure was assessed by G-LISA. The data are expressed as the means ± SD (n = 3), ^*^P < 0.05 vs. the MT + CSE group. (**C**) Co-immumoprecipitation was performed to observe the relationship between p120 and RhoA, normal goat serum was used as negative control. (**D**) The cells were transiently transfected with p120ΔRRD-GFP or MT for 48 h. Whole-cell protein was extracted and analyzed by western blot to detect p120, NF-κB-p65 and RhoA. β-actin was the loading control. The quantified results can be found as Supplementary Fig. S4A. (**E**) The cell lysates from the MT and the p120ΔRRD-GFP over-expression groups after 6 h of CSE exposure were analyzed by western blot to detect p120, NF-κB-p65 and RhoA 48 h after transfection. β-actin was the loading control. The quantified results can be found as Supplementary Fig. S4B. (**F**) The G-LISA analysis was used to detect the active RhoA after the 16HBE cells were exposed to 15% CSE in the MT and the p120ΔRRD-GFP over-expression groups. The data are expressed as the means ± SD (n = 3). (**G**) After CSE exposure, the cell lysates from the MT and the p120ΔRRD-GFP over-expression groups were measured to detect the phosphorylation of NF-κB-p65 (Ser 536) by ELISA. The data were expressed as the means ± SD.

We first detected RhoA activity in p120-knockdown 16HBE cells. Although the western blot revealed no obvious change in the total RhoA (Fig. 4A, Supplementary Fig. S3A), the G-LISA showed that RhoA activity was sharply up-regulated in the p120-knockdown group compared with the scrambled group after 15% CSE exposure (Fig. 6A). Moreover, ROCK1 increased in the p120-knockdown group (Fig. 4B, Supplementary Fig. S3B).

On the other hand, we observed that the over-expression of p120 influenced RhoA activity. The western blot revealed that both RhoA and ROCK1 were decreased in the p120 3A over-expressed groups compared with the MT groups after 15% CSE exposure (Fig. 3B, Supplementary Fig. S2B). The G-LISA showed that RhoA activity was down-regulated in the p120 3A over-expression group after 15% CSE exposure (Fig. 6B). Furthermore, we found RhoA and p120 could combine with each other in 16HBE cells by coimmunoprecipitation (Fig. 6C).

Finally, to further assess whether p120 might regulate airway inflammation via NF-κB upstream of the RhoA pathway, we conducted rescue experiments by transfecting 16HBE cells with a p120 mutant lacking the putative Rho GTPase regulatory domain (p120ΔRRD-GFP). The western blot demonstrated that the over-expression of p120ΔRRD-GFP had no effect on the expression of RhoA and NF-κB-p65 (Fig. 6D,E, Supplementary Fig. S4). The G-LISA showed that RhoA activity was not affected by the over-expression of p120ΔRRD-GFP (Fig. 6F). ELISA showed that the level of phosphorylation of NF-κB-p65 (Ser536) did not differ from that of the MT group (Fig. 6G). These results demonstrated that p120-mediated NF-κB signaling activation in airway epithelial inflammation is at least partially RhoA dependent.

### CSE-induced p120-mediated NF-κB signaling is not E-cadherin dependent

**Figure 7:**
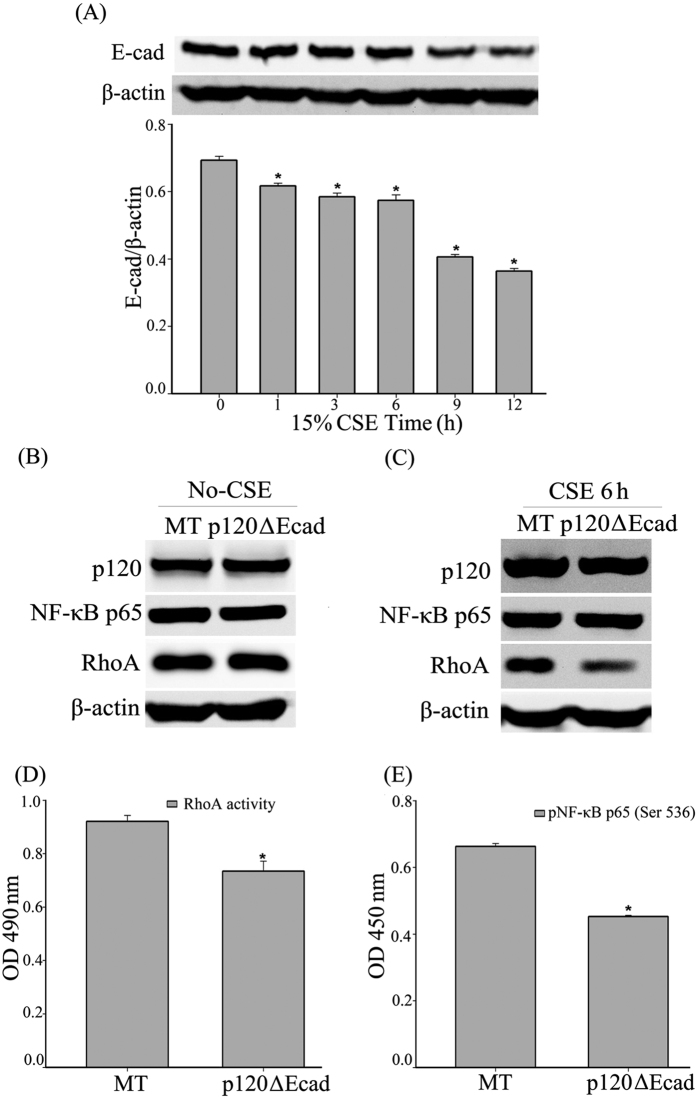
CSE-induced p120-mediated NF-κB signaling is not E-cadherin dependent. (**A**) E-cad was decreased after the 16HBE cells were exposed to CSE. The cell lysates were analyzed by western blot to detect E-cad. β-actin was the loading control. (**B**) The cells were transiently transfected with p120ΔEcad-GFP or MT for 48 h. Whole-cell protein was extracted and analyzed by western blot to detect p120, NF-κB-p65 and RhoA. β-actin was the loading control. The quantified results can be found as Supplementary Fig. S5A. (**C**) The cell lysates from the MT and the p120ΔEcad-GFP groups after 6 h of CSE exposure were analyzed by western blot to detect p120, NF-κB-p65 and RhoA 48 h after transfection. β-actin was the loading control. The quantified results can be found as Supplementary Fig. S5B. (**D**) The G-LISA results showed that RhoA activity was down-regulated in the p120ΔEcad-GFP over-expression groups compared with the MT group after 15% CSE exposure. The data are expressed as the means ± SD (n = 6), ^*^P < 0.05 vs. the MT + CSE group. (**E**) After 15% CSE exposure, the cell lysatesfrom the MT and the p120ΔEcad-GFP groups were measured to detect the phosphorylation of NF-κB-p65 (Ser 536) by ELISA. The data were expressed as the means ± SD (n = 3), ^*^P < 0.05 vs. MT + CSE group.

Many studies have reported that E-cadherin participates in RhoA regulation of NF-κB; thus, E-cadherin attracted our attention. Firstly, the western blot revealed that, after 15% CSE exposure, the expression of E-cadherin gradually decreased with the increased exposure time (Fig. 7A). Next, the plasmid p120ΔEcad-GFP, which cannot combine with E-cadherin, was transfected into the 16HBE cells. The western blot showed that the expression of RhoA and NF-κB-p65 did not change when p120ΔEcad-GFP was over-expressed in the No-CSE group (Fig. 7B, Supplementary Fig. S5A). However, RhoA expression was inhibited in the CSE exposure group (Fig. 7C, Supplementary Fig. S5B), and RhoA activity was also inhibited by the over-expression of p120ΔEcad-GFP as measured by G-LISA (Fig. 7D). The phosphorylation of NF-κB-p65 (Ser536) was attenuated after 6 h of CSE exposure in the p120ΔEcad-GFP over-expression group as measured by ELISA (Fig. 7E).

## Discussion

In the present study, we demonstrated that p120 plays a role in the cigarette smoke-induced inflammatory response presumably by regulating the RhoA-NF-κB axis. Using a human bronchial epithelial cell line as a model for the cigarette smoking-induced airway inflammatory response^25^, we demonstrated that p120 and E-cad were both decreased in CSE-exposed BECs. Additionally, NF-κB was activated, and the expression of IL-8, IL-6 and IL-1β was also increased. Transfection with the plasmid p120 3A or with p120 siRNA confirmed that p120 regulated NF-κB signaling in CSE-induced airway inflammation. Most importantly, we found that this regulation may be at least partially occurring in a RhoA/ROCK-dependent manner. p120 directly co-precipitates with RhoA in human bronchial epithelial cells. The over-expression of p120 3A inhibited RhoA activity but p120ΔRRD-GFP did not, and the silencing of p120 expression by siRNA activated RhoA. The RhoA/ROCK signaling inhibitor Y27632 greatly inhibited NF-κB-p65 (Ser536) phosphorylation and its subsequent nuclear translocation. The over-expression of mutant p120ΔEcad-GFP also inhibited both RhoA expression and activity and the phosphorylation of NF-κB-p65 (Ser536). These results demonstrate that p120-mediated NF-κB signaling activation in airway epithelial inflammation is partially RhoA dependent but not E-cad dependent.

Cigarette smoke is a major risk factor for COPD and other lung diseases, and it induces sustained NF-κB nuclear translocation, the production of inflammatory mediators, and mucus cell hypersecretion^26^. Cigarette smoke also reduces epithelial integrity^8^,^27^,^28^ and repair^28^,^29^, causing further progression of the COPD symptoms. The present study confirmed that CSE reduced the expression of the adhesion molecules p120 and E-cad in 16HBE cells. Moreover, CSE-induced NF-κB activation was accompanied by IκBα phosphorylation and IL-8, IL-6 and IL–1β production. Cigarette smoke exposure activates NF-κB signaling and leads to lung inflammation, but its underlying molecular mechanism remains poorly understood. Erk1/2 is involved in regulating NF-κB signaling in mice lungs that are exposed to cigarette smoke^26^,^30^. In addition, the RAGE-Ras–NF-kB axis has been reported to likely be associated with several smoking-related inflammatory lung diseases^31^,^32^. CSE disrupts the AJs by down-regulating p120, which modulates smoke-induced cell migration via the EGFR/Src-pathway^9^.In our study, p120 may represent a potentially novel upstream regulator of the NF-κB pathway in CSE-exposed airway inflammation.

The depletion of p120 in conditional knockout mice exhibits an inflammatory response in the intestines and epidermis as evidenced by tissue immune cell infiltration and proinflammatory cytokine release^11^,^12^,^13^. Consistent with these observations, our study provided clear evidence that p120 was down-regulated in a dose- and time-dependent manner by CSE exposure, and p120 regulated the NF-κB signaling pathway in the CSE-induced airway inflammatory response. p120 3A over-expression attenuated the CSE-stimulated phosphorylation of NF-κB-p65 (Ser536) and IL-8 mRNA expression and protein synthesis. On the contrary, p120 siRNA significantly enhanced the CSE-stimulated phosphorylation of NF-κB-p65 (Ser536) and IL-8 expression. Owing to the alternative splicing, p120 mRNAs exhibit many isoforms. In humans, this splicing produces four different p120 isoforms. Isoform 1 and 3 are the most commonly expressed^33^. Different isoforms display tissue- or cell-specific expression patterns. Isoform 1 is predominantly expressed in highly motile cells. In contrast, isoform 3, whose molecular weight 100 kD, is the most abundant in epithelial cells^34^,^35^. In our study, only p120 isoform 3 was detected in 16HBE cells and was found regulate the NF-κB activity and the expression of IL-8 after CSE exposure. This finding may to some extent be agreed with Zhang *et al*.^36^. Our results illuminate the function of p120 3A in the inflammatory process.

In our study, GTP-RhoA increased after 3 h of CSE exposure compared with other time points by G-LISA, while the total RhoA protein in the 16HBE cells showed no difference after CSE expose at different time points. All of the p120 isoforms can bind RhoA *in vitro* via a central RhoA binding site. However, only the cooperative binding of RhoA to both the central p120 domain and the alternatively spliced p120 N terminus can stabilize RhoA binding and inhibit RhoA activity^37^. Our study showed RhoA was the binding partner of p120 3A and over-expression of p120 3A inhibited RhoA expression and activity in 16HBE cells when treated with CSE. Yanagisawa^37^ reported that the existence of the N-102-234 and N-622-628 amino acid residues of p120 may be responsible for the binding to RhoA. The over-expressed p120ΔRRD-GFP that lacks its Rho GTPase-binding domain, which consists of the N-622-628 amino acid residues^38^,did not suppress CSE-induced activation of RhoA and NF-κB. This result suggests that the interaction between p120 and RhoA is necessary to prevent RhoA from activating NF-κB.

Smoke caused a time-dependent dissociation of the junctional p120/E-cad/β-ctn complexes with rapid translocation and degradation of E-cadherin. CSE reduces barrier function and the expression of the adhesion molecule E-cadherin, specifically at the cell membrane, in 16HBE cells^39^. Also, CSE exposure has been observed to decrease E-cadherin expression in cultured bronchial epithelium^8^. Kuphal, S. *et al*. reported that the loss of E-cadherin leads to the up-regulation of NF-κB activity in malignant melanoma^40^. Our results confirmed that E-cadherin was decreased in a time-dependent manner after CSE exposure in 16HBE cells. Using a mutant p120ΔEcad-GFP that cannot interact with E-cadherin^38^, we found that the over-expression of this mutant p120 could still down-regulate CSE-induced NF-κB activation. Moreover, this over-expression also inhibited the activity of RhoA. Thus, this result suggests that the role of p120 in modulating the RhoA-NF-κB pathway in airway epithelial inflammation was independent of E-cadherin.

Perez-Moreno, M. *et al*. reported that p120 affects NF-κB activation and immune homeostasis is partially through regulation of RhoA in the skin inflammation^11^,^12^. In addition, our previous studies showed that p120 was involved in inflammatory responses in a RhoA dependent manner in acute pneumonia caused by LPS^17^ and mechanical scratch caused inflammatory response^18^. Why p120-mediated NF-κB signaling activation in airway epithelial inflammation is partially RhoA dependent but not E-cad dependent? To our knowledge, RhoA is the downstream of p120 in the regulation of NF-κB signaling pathway^11^, p120 combines with E-cadherin in the cell membrane, while p120 and RhoA are co-located in the cytoplasm. A mutant p120ΔEcad-GFP could still inhibit NF-κB activation and RhoA activity, suggesting this process may occur in the cytoplasm, not in the membrane. In this situation, p120 may function as a GDI, and the binding of p120 to RhoA prevents the GEFs from switching RhoA to the GTP state independent of the binding of p120 with E-cadherin.

In summary, our data confirmed that the down-regulation of p120 can activate the NF-κB signaling pathway in CSE-exposed human bronchial epithelial cells. This process may be partially mediated in a RhoA-dependent manner. p120 directly co-precipitates with RhoA, and its down-regulation activates RhoA, which then leads to IκBα degradation and NF-κB-p65 translocation, implying that p120 has an effect on NF-κB signaling in CSE-exposure induced airway inflammation. The study of this mechanism of airway inflammation is useful because it may lead to the development of novel therapeutic strategies for a number of airway inflammatory diseases.

## Methods

### Preparation of CSE and inhibitor treatment

CSE was prepared as previously described^41^. In short, three cigarettes without filters were combusted with a modified syringe-driven apparatus. The smoke was bubbled through 25 mL of medium (100% CSE). The extract was prepared freshly and sterilized using a 0.22 mm filter and used within 30 min.

Y27632, an inhibitor of ROCK, was obtained from Sigma (St. Louis, MO, USA). The cells were starved in basal medium without growth factors for 6 h and pretreated with the above inhibitors for 12 h followed by incubation in CSE-free or CSE medium as indicated in the text.

### Cell cultures, CSE treatment, and cytotoxicity assay

The 16HBE cells, which are a simian virus 40 large T antigen transformed human bronchial epithelial cell line that retains the differentiated morphology and function of normal human bronchial epithelia^42^, were a kind gift from Dr. D.Gruenert (California Pacific Medical Center, CA, USA). The cells were cultured in Dulbecco’s Modified Eagle’s Medium (DMEM, GibcoBRL, Paisley, UK) and supplemented with 10% heat-inactivated fetal bovine serum (FBS, GibcoBRL) and antibiotics of 1% streptomycin and penicillin at 37 °C in 5% CO_2_. The cells were cultured in the presence and absence of CSE (1%, 5%, 10%, 15% and 20%) for different time points.

To investigate the effects of CSE on cell activity, the 16HBE cells were exposed to CSE (0, 10%, 20%, 30%, 40% and 50%) for 24 h. The cytotoxicity assay has been described previously^43^. When the cells reached 80–90% confluence, they were rinsed two times with PBS and incubated in serum-free culture medium with or without the different concentrations of CSE (according to the MTT assay).

### Immunofluorescent analysis

The cells were fixed with 4% paraformaldehyde, permeabilized with 0.3% Triton X-100 and incubated with primary antibodies at 4 °C overnight. The antibodies directed against NF-κB-p65 were obtained from Cell Signaling Technology (San Jose, CA, USA) and used according to the manufacturer’s instructions. The nuclei were stained with DAPI. The images were photographed with an Eclipse FV500 confocal laser scanning microscopy system (Olympus, Tokyo, Japan) with the appropriate filter sets.

### Enzyme-linked immune sorbent assay

After stimulation, the cell lysate supernatants were collected and measured for IL-8, IL-6, IL-1β and NF-κB-p65 (Ser536) by ELISA using a commercial kit from R&D Systems (Minneapolis, MN, USA) according to the manufacturer’s protocol. The absorbance corresponding to the phosphorylation of NF-κB-p65 (Ser536) was measured at 450 nm, and the concentrations of IL-8, IL-6 and IL-1β were determined using an internal standard curve.

### Quantitative real-time PCR

Total RNA was extracted with the TRIzol Reagent (Invitrogen Carlsbad, CA, USA), and the concentration was measured using an ultraviolet (UV) spectrophotometer (UV-1201; Shimadzu Corporation, Kyoto, Japan). Reverse transcription (RT) was performed as described previously^43^. Real-time PCR was conducted using the SYBR-Green PCR kit (Takara, Osaka, Japan) in a Rotor-Gene 3000 machine (Corbett Life Science, Sydney, Australia). The quantitative analysis of the transcription of p120 and IL-8 was described previously^16^. Each reaction was performed in a 25 μL volume containing 2 μL of cDNA, 0.5 μL of 10 μM per each primer, and 12.5 μL of the 2 × SYBR-Green mixture. p120: For: 5′-GGA CAC CCT CTG ACC CTC G-3′, Rev: 5′-GCT TGC TAA ACT TCC TCG CTC-3′, product of 122bp. IL-8: For: 5′- ACA CTG CGC CAA CAC AGA AAT TA-3′, Rev: 5′ -TTT GCT TGA AGT TTC ACT GGC ATC -3′, product of 185bp.

### Coimmunoprecipitation and Western blot analysis

Coimmunoprecipitation, cytoplasmic and nuclear extracts, and Western blot analysis were performed as described previously^43^.

### RNA interference

The human p120 small interfering RNA (p120 siRNA) oligonucleotide was purchased from Santa Cruz Biotechnology, Inc. (Dallas, TX, USA). The cells were seeded onto a 6-well plate at 30% confluence in complete medium and transfected with p120 siRNA using Lipofectamine 2000 according to the manufacturer’s recommended procedure. The nonsilencing siRNA (scrambled) was used as the control.

### Plasmids and transient transfection

The plasmid RcCMV mp120-3A was generously provided by Professor Enhua Wang. The plasmids p120ΔRRD-GFP and p120ΔEcad-GFP were generously provided by Professor Mirna Perez-Moreno^38^. To achieve a transient gene transfection, the cells were used in the exponential phase of growth, and the transfection was performed using Lipofectamine 2000 according to the manufacturer’s recommendation and the method described by Tucker *et al*.^44^ with minor modifications.

### RhoA activation assay

The RhoA G-LISA^®^ kit was purchased from Cytoskeleton Inc. (Cat. #BK124; Denver, CO, USA). According to the protocol, the 16HBE cells were collected in ice-cold cell lysis buffer and immediately snap frozen in liquid nitrogen and stored at −80 °C to minimize GTP hydrolysis. An aliquot was set aside for protein concentration determination using the Precision Red^TM^ Advanced Protein Assay Reagent supplied with the kit. Equal protein aliquots were added to the individual wells in an eight-well strip coated with an appropriate RBD, and the plates were incubated on a cold orbital microplate shaker at 400 rpm at 4 °C for exactly 30 min. The strips were washed and incubated with an anti-RhoA primary antibody followed by an HRP-conjugated secondary antibody, and the HRP detection reagent supplied with the kit was used. The signal was read by measuring the absorbance at 490 nm using a microplate spectrophotometer (SpectraMax 340 Microplate Reader; Molecular Devices). The samples from at least three independent experiments were assayed in triplicate.

### Statistical analysis

The results are expressed as the means ± SD of experiments repeated at least three independent times. The statistical significance was determined using SPSS 19.0 software. The data were evaluated with one-way analysis of variance (ANOVA) combined with a post hoc analysis (Fisher’s PLSD). A value of *p *< 0.05 was considered significant.

## Additional Information

**How to cite this article**: Zhang, C. *et al*. Cigarette smoke extract-induced p120-mediated NF-κB activation in human epithelial cells is dependent on the RhoA/ROCK pathway. *Sci. Rep.* **6**, 23131; doi: 10.1038/srep23131 (2016).

## Supplementary Material

Supplementary Information
