# Supplementary material for: Cigarette smoke extract-induced p120-mediated NF-κB activation in human epithelial cells is dependent on the RhoA/ROCK pathway
Source: Sci Rep. 2016 Sep 2;6:23131. doi: 10.1038/srep23131 (PMC5009380; doi:10.1038/srep23131)
Supplement: Supplementary Information [file srep23131-s1.pdf]

Cigarette smoke extract-induced p120-mediated NF-κB activation in human epithelial cells is dependent on the RhoA/ROCK pathway

Chao Zhang[1,+] , Shenghui Qin[1,+], Lingzhi Qin[1], Liwei Liu[1], Wenjia Sun[1], Xiyu Li[1], Naping Li[1], Renliang Wu[1], Xi Wang[1,*]

[1]Institute of Pathology, Tongji Hospital, Tongji Medical College, Huazhong University of Science and Technology, Wuhan 430030, China

[+]These authors contributed equally to this work

Correspondence and requests for materials should be addressed to X.W. (xwang@mails.tjmu.edu.cn)

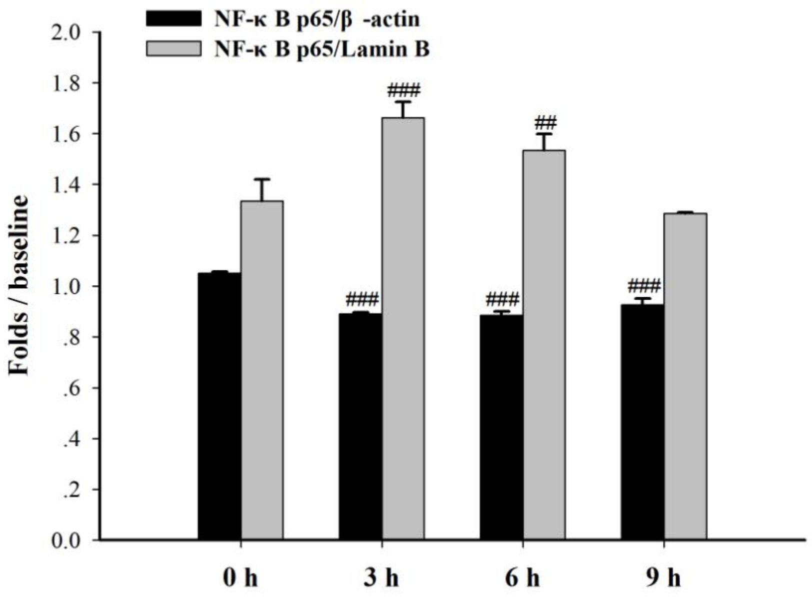

Figure S1 | The quantified results of figure 2C and expressed as fold of loading control. The data are expressed as the means ± SD (n = 3), $^{\#\#}P$<0.01, $^{\#\#\#}P$< 0.001 vs. CSE 0 h groups.

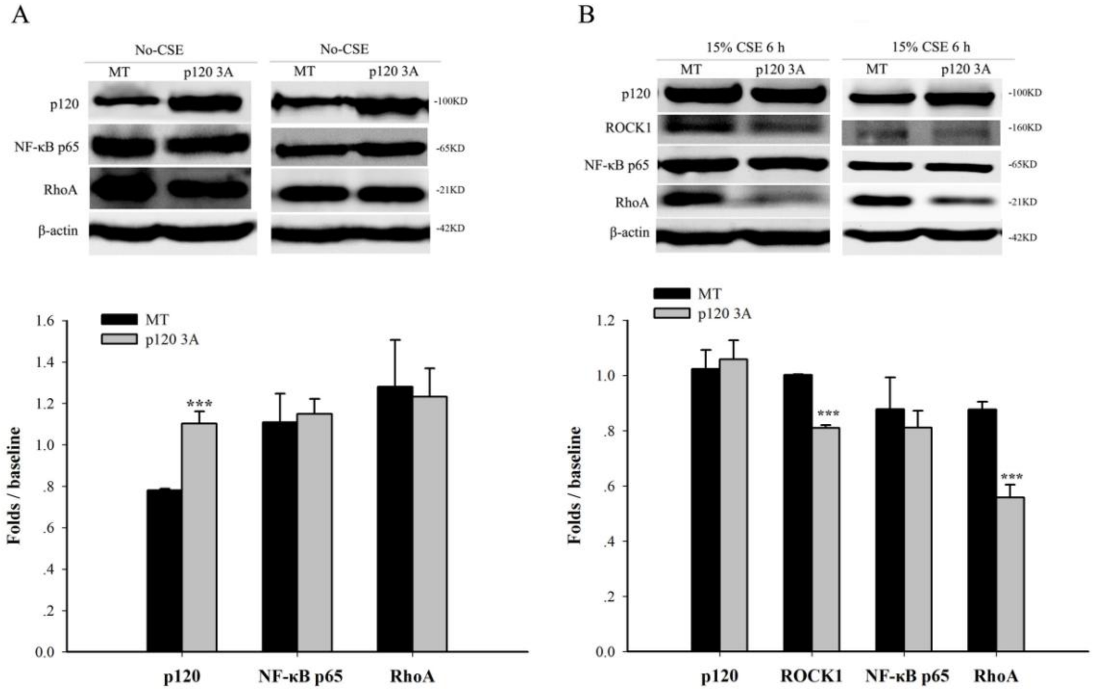

Figure S2 | (A) The representative images and quantified results of figure 3A. The data are expressed as the means ± SD (n = 3), ***P<0.001 vs. the MT group. (B) The representative images and quantified results of figure 3B. The data are expressed as the means ± SD (n = 3), ***P<0.001 vs. the MT group.

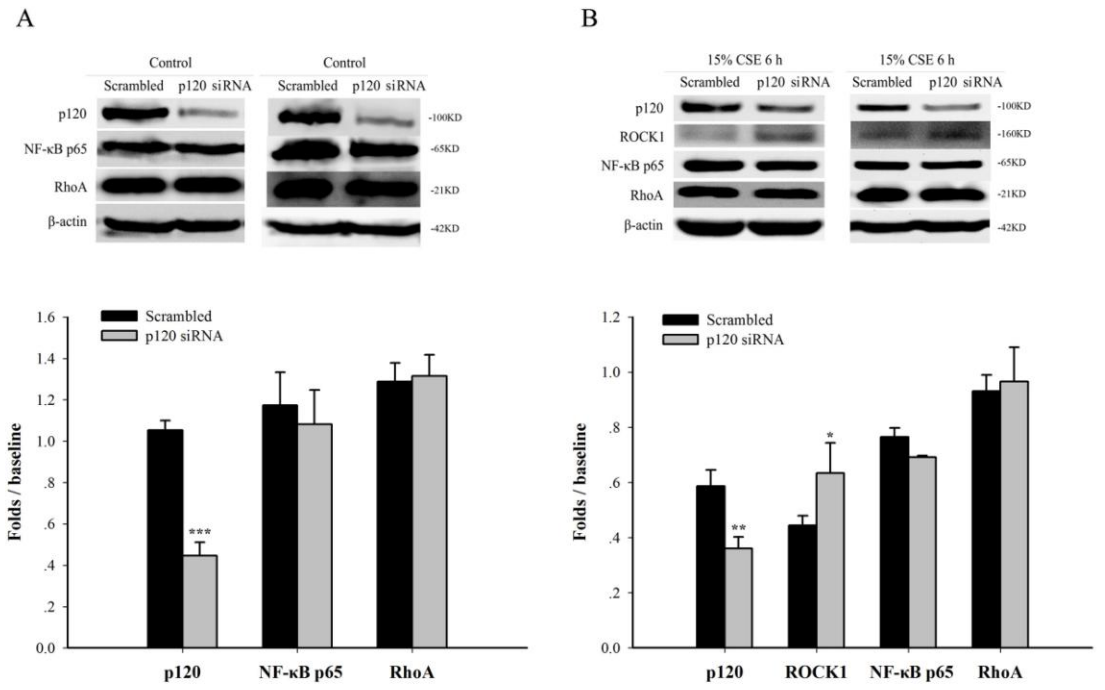

Figure S3 | (A) The representative images and quantified results of figure 4A. The data are expressed as the means ± SD (n = 3), ***P<0.001 vs. the scrambled group. (B) The representative images and quantified results of figure 4B. The data are expressed as the means ± SD (n = 3), *P<0.05, **P<0.01 vs. the scrambled group.

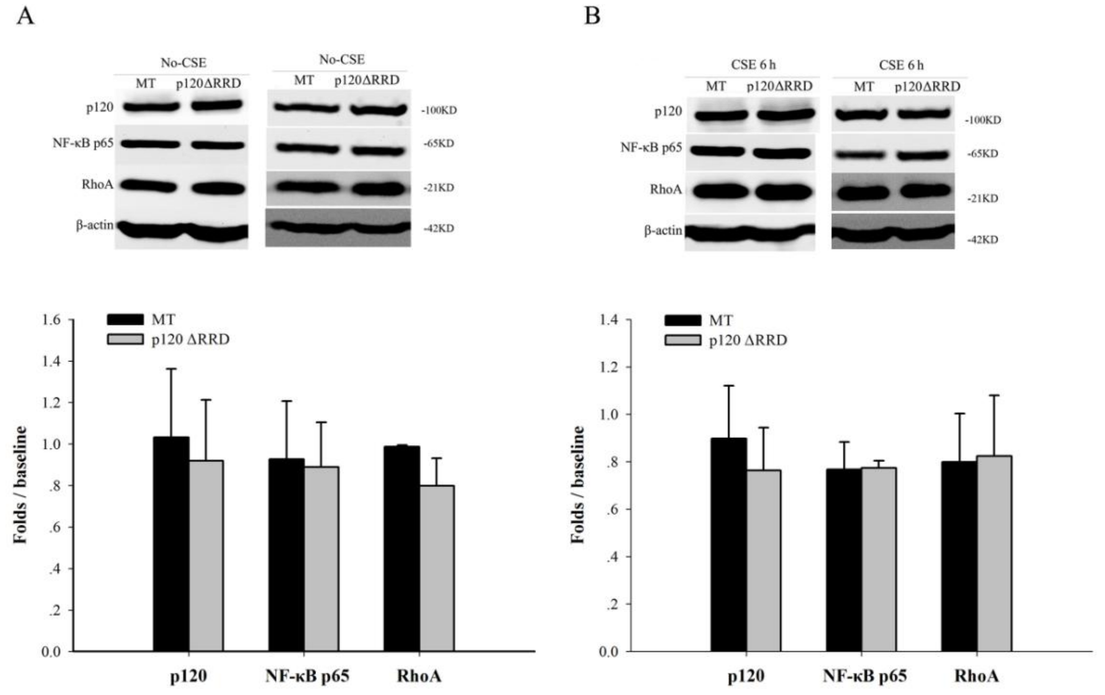

Figure S4 | (A) The representative images and quantified results of figure 6D. The data are expressed as the means ± SD (n = 3). (B) The representative images and quantified results of figure 6E. The data are expressed as the means ± SD (n = 3).

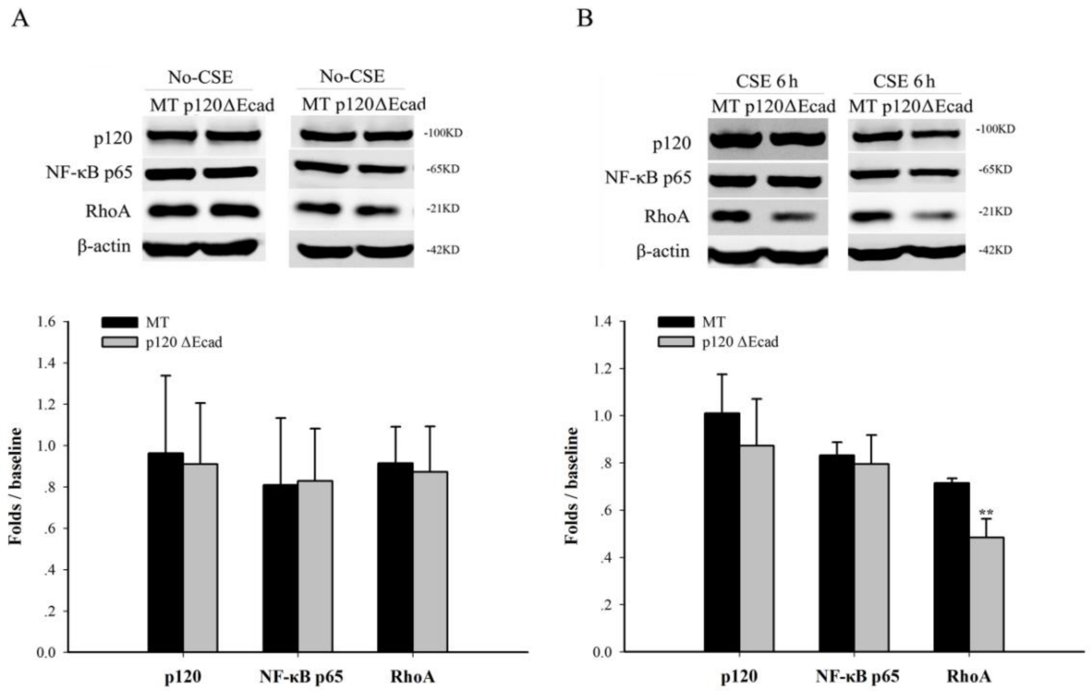

Figure S5 | (A) The representative images and quantified results of figure 7B. The data are expressed as the means ± SD (n = 3). (B) The representative images and quantified results of figure 7C. The data are expressed as the means ± SD (n = 3), **P<0.01 vs. the MT group.
